# Diversity and characterization of temperate bacteriophages induced in *Pasteurella multocida* from different host species

**DOI:** 10.1186/s12866-021-02155-9

**Published:** 2021-03-30

**Authors:** Rezheen F. Abdulrahman, Robert L. Davies

**Affiliations:** 1 Institute of Infection, Immunity and Inflammation, College of Medical, Veterinary and Life Sciences, Sir Graeme Davies Building, University of Glasgow, 120 University Place, Glasgow, G12 8TA UK; 2 Pathology and Microbiology Department, Collage of Veterinary Medicine, University of Duhok, Kurdistan Region, Iraq

**Keywords:** *Pasteurella multocida*, Bacteriophage diversity, Bacteriophage morphology

## Abstract

**Background:**

Bacteriophages play important roles in the evolution of bacteria and in the emergence of new pathogenic strains by mediating the horizontal transfer of virulence genes. *Pasteurella multocida* is responsible for different disease syndromes in a wide range of domesticated animal species. However, very little is known about the influence of bacteriophages on disease pathogenesis in this species.

**Results:**

Temperate bacteriophage diversity was assessed in 47 *P. multocida* isolates of avian (9), bovine (8), ovine (10) and porcine (20) origin. Induction of phage particles with mitomycin C identified a diverse range of morphological types representing both *Siphoviridae* and *Myoviridae* family-types in 29 isolates. Phage of both morphological types were identified in three isolates indicating that a single bacterial host may harbour multiple prophages. DNA was isolated from bacteriophages recovered from 18 *P. multocida* isolates and its characterization by restriction endonuclease (RE) analysis identified 10 different RE types. Phage of identical RE types were identified in certain closely-related strains but phage having different RE types were present in other closely-related isolates suggesting possible recent acquisition. The host range of the induced phage particles was explored using plaque assay but only 11 (38%) phage lysates produced signs of infection in a panel of indicator strains comprising all 47 isolates. Notably, the majority (9/11) of phage lysates which caused infection originated from two groups of phylogenetically unrelated ovine and porcine strains that uniquely possessed the *toxA* gene.

**Conclusions:**

*Pasteurella multocida* possesses a wide range of *Siphoviridae-* and *Myoviridae-*type bacteriophages which likely play key roles in the evolution and virulence of this pathogen.

**Supplementary Information:**

The online version contains supplementary material available at 10.1186/s12866-021-02155-9.

## Background

*Pasteurella multocida* is a Gram-negative commensal bacterium which resides in the upper respiratory tract of mammals and birds [1]. The organism is responsible for various economically important diseases in a wide range of domestic animal species [1]. It causes fowl cholera in poultry [1–3], haemorrhagic septicaemia in cattle and water buffalo [4], atrophic rhinitis of pigs [5–7], pneumonia in cattle, sheep and pigs [5, 8, 9], snuffles of rabbits [10] and septicaemia in neonatal lambs [11, 12]. Infection with *P. multocida* may also occur in humans following dog and cat bites or scratches [13–15]. *Pasteurella multocida* possesses a broad range of virulence factors that play important roles in disease pathogenesis and which include capsule, lipopolysaccharide and outer membrane proteins [16]. Specific virulence-associated genes also thought to be involved in the pathogenesis of *P. multocida* include *tbpA*, *pfhA, toxA*, *hgbB*, *hgbA*, *nanH*, *nanB*, *sodA*, *sodC*, *oma87*and *ptfA* [16, 17].

Bacteriophages play important roles in the evolution of bacteria and in the emergence of new strains; in particular, they are involved in the transfer of a wide range of virulence genes and can strongly influence the pathogenic potential of bacteria [18–24]. Temperate bacteriophages are those which become integrated into the bacterial chromosome to become a prophage [25] and, in the process, may induce a change in the phenotype of the infected bacterium in a process called lysogenic conversion [26]. Those temperate bacteriophages that carry virulence genes may transform a bacterial host from a commensal to a pathogenic strain and can contribute significantly to bacterial virulence [27–30]. Toxins represent an important class of virulence genes that are transferred by bacteriophages [18, 19, 31], but phage also encode genes involved in adhesion, colonisation, immune evasion and serum resistance, as well as the expression of surface-exposed antigens [18, 25, 28, 31, 32]. The presence of these genes within phage genomes suggests that there is an evolutionary advantage for the bacteriophages to carry such genes, perhaps due to enhanced replication of bacteria carrying these virulence determinants [31].

Very little is known about the temperate bacteriophages of *P. multocida* and their potential role in the virulence and evolution of this pathogen. Bacteriophages have been described in *P. multocida* and used as a typing method in epidemiological studies [33, 34]. The morphology of temperate phage in a small number of *P. multocida* isolates has been described and a diverse group of phage belonging to the *Myoviridae*, *Siphoviridae* and *Podoviridae* families identified [35]. Certain porcine strains of *P. multocida* responsible for atrophic rhinitis produce a mitogenic toxin (PMT) that is encoded by the *toxA* gene; notably, *toxA* is associated with a lysogenic *Siphoviridae*-type bacteriophage [36]. However, *toxA* has also more recently been identified in ovine *P. multocida* isolates [17, 37, 38], suggesting a role for bacteriophages in the horizontal transfer of *toxA* from porcine to ovine strains.

The diversity of temperate bacteriophages associated with *P. multocida* strains of known genetic relatedness and originating from different host species and disease syndromes has not previously been assessed. In the present study, we aimed to investigate the presence and diversity of temperate bacteriophages within a collection of *P. multocida* strains recovered from avian, bovine, ovine and porcine hosts with a longer-term view of assessing whether bacteriophages are contributing to the evolution of *P. multocida* in these different host species. To achieve these aims, bacteriophages were induced in 47 *P. multocida* isolates carefully selected to represent specific phylogenetic lineages, host species of origin, disease types, capsular serotypes and OMP-types. Bacteriophages were induced using mitomycin C which has commonly been used for bacteriophage induction in a wide range of bacterial species [39–42] including *P. multocida* [33, 36, 43]. The induced bacteriophages were subsequently characterized by assessing their morphologies using transmission electron microscopy (TEM) and examining their genetic relatedness using DNA restriction endonuclease (RE) analysis. In addition, the host range of selected bacteriophages was assessed against a panel of indicator strains comprising all 47 *P. multocia* isolates.

## Results

### Bacteriophage induction

Mitomycin C is commonly used to induce temperate bacteriophages in bacteria and typically causes lysis of the bacteria which results in clearing of the bacterial suspension over time. However, the rate and degree of lysis can vary according to the properties and density of the induced phage [44, 45]. To identify the optimum concentration of mitomycin C required to induce temperate bacteriophages in *P. multocida* isolates, seven different concentrations were tested against eight strains of avian (PM144, PM246), bovine (PM564, PM632), ovine (PM966, PM982) and porcine (PM684, PM734) origin. These strains represent various capsular serotypes, OMP types and multilocus sequence types (STs) and their properties are shown in Table 1. The OD_600_ values were plotted against time (h) for the control and seven different mitomycin C concentrations for each isolate and three general patterns were observed; these were designated complete lysis, partial lysis or reduced growth, and no lysis or no reduced growth (Fig. S1). Isolates PM684 and PM982 were highly sensitive to mitomycin C treatment compared to the other isolates and underwent rapid and complete lysis by 0.2 μg/ml mitomycin C (Fig. S1A & B). The growth of isolates PM246, PM632, PM734 and PM966 was progressively reduced as the mitomycin C concentration increased; however, complete lysis, as observed with PM684 and PM982, was not achieved even at the highest concentration of mitomycin C (Fig. S1C-F). Isolates PM144 and PM564 exhibited little or no effect from mitomycin C although the final OD_600_ values, even for the controls, were not as high as for the other six isolates (Fig. S1G & H). Overall, the results demonstrated that final concentrations of 0.2, 0.5, 1.0 and 2.0 μg/ml can be used for assessing induction of temperate phage in *P. multocida*. However, a concentration of 0.2 μg/ml mitomycin C was selected as optimum because this concentration was sufficient to cause complete and rapid bacterial cell lysis in isolates PM684 and PM982.

**Table 1:** Details of 47 *P. multocida* isolates

Isolate^a^	Host species	Disease syndrome	Isolation site	MLST lineage	ST^b^	Capsular type	OMP- type^c^	*toxA* gene	Indicator strain group (ISG)
PM316	Bovine	Pneumonia	Lung	A	1	A	1.1	ND	1a
PM564	Bovine	Pneumonia	Lung	A	1	A	2.1	ND^d^	1a
PM344	Bovine	Pneumonia	Lung	A	3	A	3.1	ND	2a
PM632	Bovine	Pneumonia	Lung	A	4	A	4.1	ND	2b
PM666	Porcine	Pneumonia	Lung	A	3	A	2.1	–	2b
PM116	Porcine	Pleuropneumonia	Lung	A	3	A	3.1	–	2b
PM966	Ovine	Pneumonia	Lung	B	16	A	1.1	–	
PM382	Porcine	Respiratory problems	Lung	C	13	A	4.1	–	
PM706	Porcine	Pneumonia	Lung	C	13	UT	4.1	–	
PM2	Ovine	Severe peritonitis	–	C	17	F	2.1	–	
PM8	Ovine	Asymptomatic	Vagina	C	17	F	2.1	–	
PM246	Avian	Septicaemia	Viscera	C	25	F	2.2	ND	
PM994	Ovine	Pneumonia	Lung	C	12	F	1.1	–	
PM148	Avian	Eye infection	Eye	C	12	F	2.2	ND	
PM104	Avian	Septicaemia	Liver/spleen	D	28	A	4.1	ND	
PM86	Avian	Fowl cholera	Pleura	D	15	A	3.1	ND	3a
PM934	Porcine	Pneumonia	Lung	D	15	A	5.1	–	3a
PM954	Porcine	Pneumonia	Lung	D	15	A	5.1	–	3a
PM486	Bovine	Pneumonia	Lung abscess	D	9	A	9.1	ND	3a
PM172	Avian	Septicaemia	Lung	D	26	A	3.1	ND	3a
PM302	Bovine	Rhinitis + others	Nasal swab	E	6	A	5.3	ND	3b
PM144	Avian	Septicaemia	Lung/liver	E	21	A	1.1	ND	
PM402	Bovine	Pneumonia	Lung	E	5	A	5.1	ND	
PM122	Ovine	Pneumonia	Lung	ND	ND	D	3.1	+	
PM964	Ovine	Pneumonia	Lung	E	18	D	3.1	+	
PM982	Ovine	Pneumonia	Lung	E	18	D	3.1	+	
PM986	Ovine	Pneumonia	Lung	E	18	D	3.1	+	
PM988	Ovine	Pasteurellosis	Lung	ND	ND	D	3.1	+	
PM990	Ovine	Pneumonia	Lung	ND	ND	D	3.1	+	
PM54	Porcine	Pneumonia	Lung	F	10	A	1.1	–	4
PM734	Porcine	Pneumonia	Lung	F	10	A	1.1	–	4
PM820	Porcine	Pneumonia	Lung	F	10	A	1.1	–	4
PM850	Porcine	Pneumonia	Lung	F	10	A	1.1	–	4
PM200	Avian	Pneumonia	Lung	F	10	A	1.2	ND	
PM336	Bovine	Pneumonia	Lung	F	7	A	6.1	ND	1b
PM684	Porcine	Suspected atrophic rhinitis	Nasal swab	G	11	A	6.1	+	5
PM918	Porcine	Pneumonia	Lung	G	11	A	6.1	+	5
PM926	Porcine	Pneumonia	Lung	ND	ND	A	6.1	+	5
PM40	Porcine	–	–	ND	ND	A	6.2	+	5
PM716	Porcine	Suspected atrophic rhinitis	Nasal swab	G	11	D	4.1	+	
PM848	Porcine	Pneumonia	Lung	G	11	D	4.1	+	
PM696	Porcine	Suspected atrophic rhinitis	Nasal swab	G	11	D	6.1	+	
PM714	Porcine	Pneumonia	Lung	G	11	D	6.1	–	
PM762	Porcine	Atrophic rhinitis	Turbinate	G	11	D	6.1	+	
PM890	Porcine	Suspected atrophic rhinitis	Nasal swab	G	11	D	6.1	+	
PM226	Avian	Pneumonia/death	Lesion	G	11	D	13.1	–	
PM82	Avian	Swollen heads	Peritoneal	H	32	A	7.1	ND	

^a^ Isolates are arranged by order of MLST lineage (column 5; Fig. 5); ^b^ ST: sequence types (Davies et al.*,* unpublished; http://pubmlst.org/pmultocida_multihost); ^c^ OMP-types for bovine, ovine, porcine and avian isolates have been defined previously [9, 46–49] and are not equivalent, i.e. bovine OMP-type 1.1 is not as same as porcine OMP-type 1.1, etc.; ^d^ ND: not determined

**Fig. 1:**
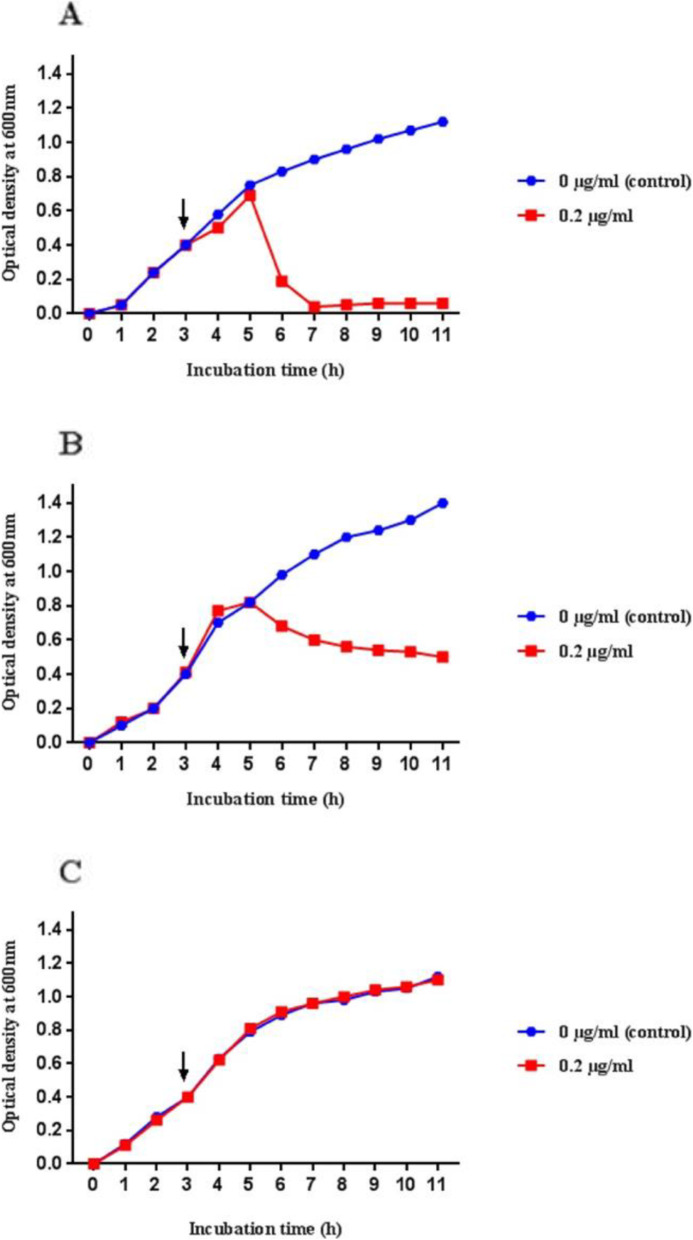
Phage induction profiles showing (**a**) complete lysis, (**b**) partial lysis or reduced growth and (**c**) no lysis or no reduced growth with 0.2 μg/ ml mitomycin C. Mitomycin C was added once the OD_600_ reached 0.4 and the OD_600_ was plotted against time (h). The induction profile for each isolate was generated by comparing the OD_600_ with the control (no mitomycin C) over 11 h. The arrows indicate the points at which the mitomycin C was added. Graphs were created using GraphPad Prism 7

The induction profiles of 47 isolates of *P. multocida* from the four host species and representing various capsular serotypes, OMP-types, and STs were subsequently generated first using 0.2 μg/ml mitomycin C. In the absence of lysis with the 0.2 μg/ml mitomycin C, the effect of using higher concentrations of 0.5, 1.0 and 2.0 μg/ml was assessed. In this way, the 47 isolates were assigned to one of the three induction profiles described above: complete lysis, partial lysis or reduced growth, and no lysis or no reduced growth. An example of each induction profile type is shown in Fig. 1. Taking the 47 isolates into account, complete lysis was characterized by a rapid decrease in optical density which typically attained a final OD_600_ value of < 0.4 (Fig. 1a); partial lysis or reduced growth was characterized by a gradual decrease in optical density or flattening of the curve which typically attained a final OD_600_ value of > 0.4 (Fig. 1b); and no lysis or no reduced growth was characterized by no or little observable difference between the mitomycin C-treated cultures and the controls (Fig. 1c). The induction profiles assigned to the 47 isolates are summarised in Table S1. The majority (37/47) of the strains were either completely or partially lysed with 0.2 μg/ml mitomycin C, although a proportion of isolates required exposure to higher concentrations of mitomycin C (ranging from 0.5 to 2.0 μg/ml) to induce either partial or complete lysis. Fifteen (32%) isolates exhibited complete lysis, 22 (47%) were partially lysed or showed reduced growth, and 10 (21%) showed no lysis or no reduced growth even after exposure to 2.0 μg/ml mitomycin C for 24 h. Ten isolates that initially exhibited no signs of lysis with 0.2 μg/ml mitomycin C were subsequently shown to be induced by higher concentrations of mitomycin C (Table S1).

### Bacteriophage morphology by TEM

**Fig. 2:**
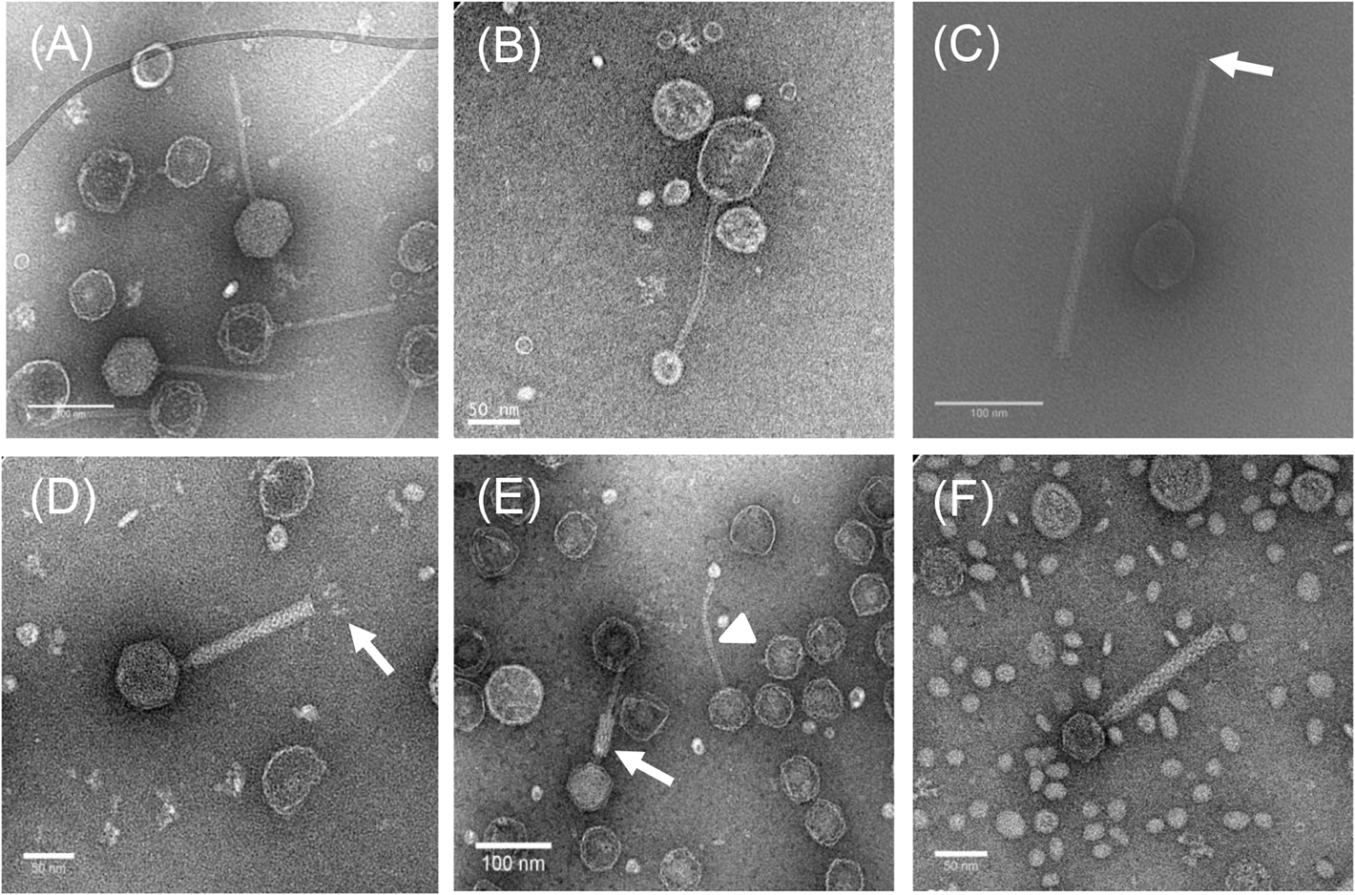
Electron micrographs of phage induced in *P. multocida* isolates. Differences were observed in the morphology of phage induced in *P. multocida* isolates. *Siphoviridae-like* phage possessed hexagonal (**a**) or elongated (**b**) capsids connected to flexible tails. Differences were also observed in the morphology of *Myoviridae-like* phage which possessed extended sheaths and blunt ends (**c**; arrow), extended sheaths and prominent base plates and tail fibres (**d**; arrow) or contracted sheaths and blunt tail ends (**e**; arrow). In some cases, *Myoviridae*- and *Siphoviridae-*type phage were both induced in the same bacterial hosts (**e**; arrow and arrowhead). *Myoviridae*-type phage were also identified in a sub-set of isolates which possessed unique, small hexagonal capsids (**f**)

Triplicate samples of cultures representing each isolate were subjected to mitomycin C treatment and the cultures examined by TEM. Overall, phage particles were identified in 29 *P. multocida* isolates that were treated with mitomycin C (Table 2). Bacteriophages were identified in 14/15 (93%) isolates that underwent complete lysis, in 15/22 (68%) that underwent partial lysis or reduced growth, and in none of the 10 isolates that showed no evidence of lysis or reduced growth (Table S1). Based on their tail morphologies, the phage particles identified belonged to the *Siphoviridae* and *Myoviridae* families of the order Caudovirales. *Siphoviridae*-like phage were induced exclusively in 18 (62.1%) *P. multocida* isolates whereas *Myoviridae*-like phage were induced in six (20.7%) isolates; however, both *Siphoviridae-* and *Myoviridae-*type phage were identified in three (10.3%) isolates and tail-less capsids were identified in two (6.9%) isolates (Table 2). Variation was observed in the morphology of the *Siphoviridae*-type phage; the shape and size of both the capsid and tail of phage varied between certain isolates. Thus, these phage possessed either hexagonal or elongated capsids and the head size varied from 50 to 80 nm long and 55 to 65 nm wide. The capsids were connected to flexible tails approximately 110 to 242 nm long and 6 to 9 nm in diameter (Fig. 2a & b; Table 2). *Myoviridae*-type phage were characterized by the possession of hexagonal capsids 59 to 63 nm long and 51 to 56 nm wide and long contractile tails with either extended or contracted sheaths; the tails were approximately 150 nm long and 16 to 18 nm wide (Table 2). Variation was also observed in the tail ends: some had blunt ends (Fig. 2c, arrow) whereas others had prominent base plates and tail fibres (Fig. 2d, arrow). Both *Siphoviridae*- and *Myoviridae*-type phage were identified in isolates PM86, PM684 and PM850 indicating that a single host may harbour multiple prophages (Fig. 2e, arrows; Table 2). Notably, the five isolates PM86, PM172, PM486, PM934 and PM954 contained a distinct *Myoviridae*-type phage (Fig. 2f). These phage particles (discussed further below) were unique in that they possessed relatively small hexagonal capsid heads that were only 38 to 39 nm long and 37 nm wide; their tails were approximately 190 nm long and 18 nm wide (Table 2). Hexagonal, apparently tail-less, capsids approximately 65 nm diameter were observed in isolates PM762 and PM890 (images not shown).

**Table 2:** Properties of temperate bacteriophages identified in 29 *P. multocida* isolates

Isolate	Lysis type^a^	No of phage types (TEM)	Family type^b^	Head size (nm)^c^	Tail size (nm)^d^	Host range group (HRG)	Phage DNA	RE type
PM666	Partial	1	*Siphoviridae*	50 × 55	132 × 8	–	–	–
PM116	Partial	1	*Siphoviridae*	50 × 56	138 × 8	–	–	–
PM966	Partial	1	*Siphoviridae*	52 × 58	110 × 8	–	–	–
PM382	Complete	1	*Myoviridae*	63 × 55	147 × 16	–	+	A
PM86	Partial	2	*Myoviridae & Siphoviridae*	38 × 37/63 × 55	190 × 18/138 × 7	–	+	J
PM934	Partial	1	*Myoviridae*	38 × 37	190 × 18	–	+	B
PM954	Partial	1	*Myoviridae*	38 × 37	190 × 18	–	+	B
PM486	Partial	1	*Myoviridae*	38 × 37	190 × 18	–	+	B
PM172	Partial	1	*Myoviridae*	39 × 37	190 × 18	–	+	B
PM122	Complete	1	*Siphoviridae*	66 × 67	142 × 8	I	+	C
PM964	Complete	1	*Siphoviridae*	62 × 55	141 × 8	I	+	C
PM982	Complete	1	*Siphoviridae*	63 × 61	146 × 9	I	+	C
PM986	Complete	1	*Siphoviridae*	64 × 60	149 × 8	I	+	C
PM988	Complete	1	*Siphoviridae*	64 × 55	136 × 8	I	+	C
PM54	Partial	1	*Siphoviridae*	54 × 54	142 × 9	–	–	–
PM820	Partial	1	*Siphoviridae*	54 × 54	142 × 9	–	–	–
PM850	Complete	2	*Myoviridae & Siphoviridae*	59 × 51/52 × 59	148 × 16/134 × 8	III	+	D
PM200	Complete	1	*Siphoviridae*	63 × 57	138 × 7	–	–	–
PM336	Complete	1	*Siphoviridae*	80 × 59	154 × 7	IV	+	E
PM684	Complete	2	*Myoviridae & Siphoviridae*	63 × 52/59 × 57	148 × 15/125 × 8	IIa	+	F
PM918	Complete	1	*Siphoviridae*	55 × 65	242 × 8	IIb	+	G
PM926	Complete	1	*Siphoviridae*	66 × 56	149 × 8	IIb	+	G
PM40	Complete	1	*Myoviridae*	60 × 56	144 × 16	IIc	+	H
PM848	Complete	1	*Siphoviridae*	63 × 61	143 × 9	–	+	I
PM696	Partial	1	*Siphoviridae*	60 × 54	110 × 7	–	–	–
PM714	Partial	1	*Siphoviridae*	55 × 56	107 × 6	–	–	–
PM762	Partial	1	Tail-less phage	66 × 54	–	–	–	–
PM890	Partial	1	Tail-less phage	65 × 60	–	–	–	–
PM226	Partial	1	*Siphoviridae*	54 × 56	126 × 8	–	–	–

^a^ Complete lysis when the final OD_600_ was 0.4 or less; partial lysis when the OD_600_ was above 0.4; little or no lysis when the OD_600_ values in control and mitomycin C treated cultures remained constant after being treated with mitomycin C for 8 h of incubation. ^b, c^ and ^d^ based on TEM

### Restriction endonuclease analysis of phage DNA

**Fig. 3:**
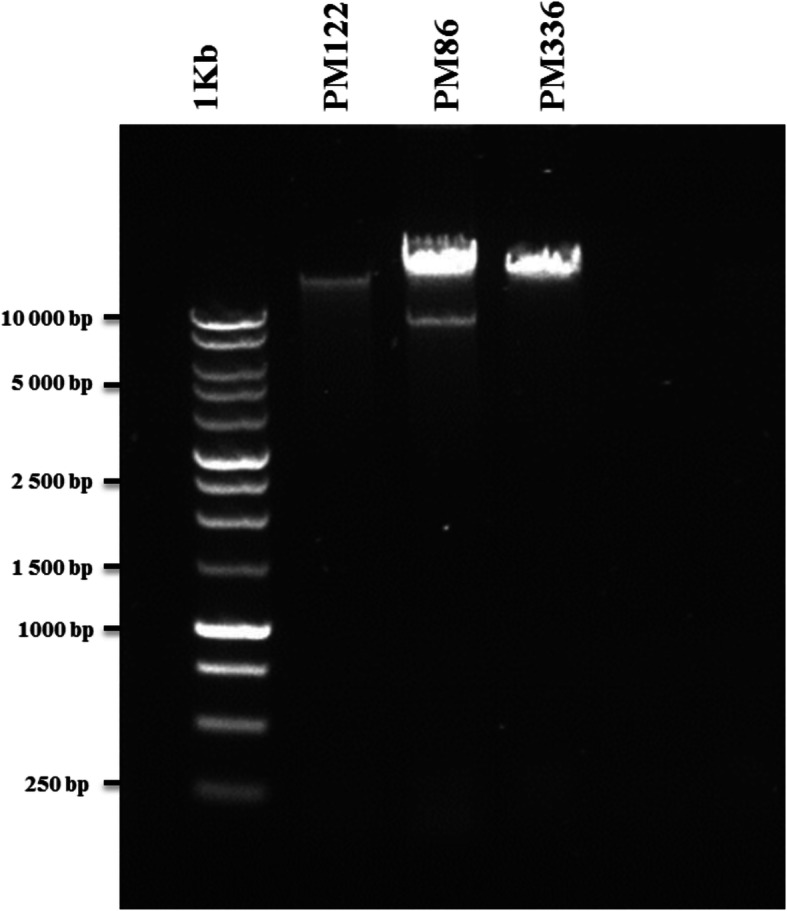
Phage DNA isolated from temperate bacteriophages of *P. multocida*. Varying amounts of DNA were recovered from different isolates (PM122 and PM336) and, in some cases (PM86), two DNA bands were present. Two bands of DNA were isolated from five *P. multocida* isolates (PM86, PM172, PM486, PM934 and PM954) and suggested the presence of more than one phage type

**Fig. 4:**
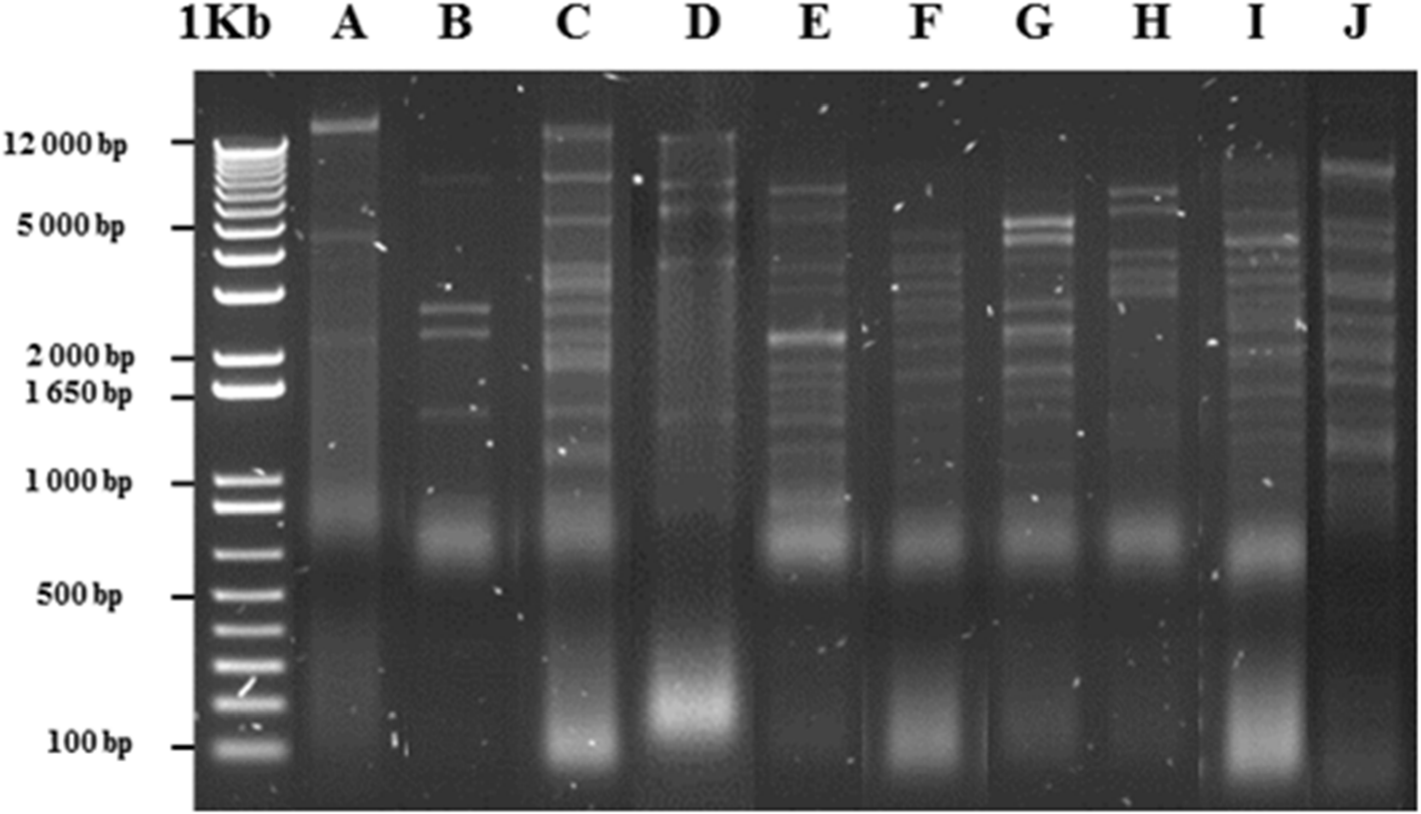
Restriction endonuclease (RE) types of phage DNA isolated from *P. multocida*. Phage DNA was digested with HindIII. Ten unique RE types were identified in 18 different phage DNA samples as follows: type A (ФPM382), type B (ФPM172, ФPM486, ФPM934 and ФPM954), type C (ФPM122, ФPM964, ФPM982, ФPM986 and ФPM988), type D (ФPM850), type E (ФPM336), type F (ФPM684), type G (ФPM918 and ФPM926), type H (ФPM40), type I (ФPM848) and type J (ФPM86)

**Fig. 5:**
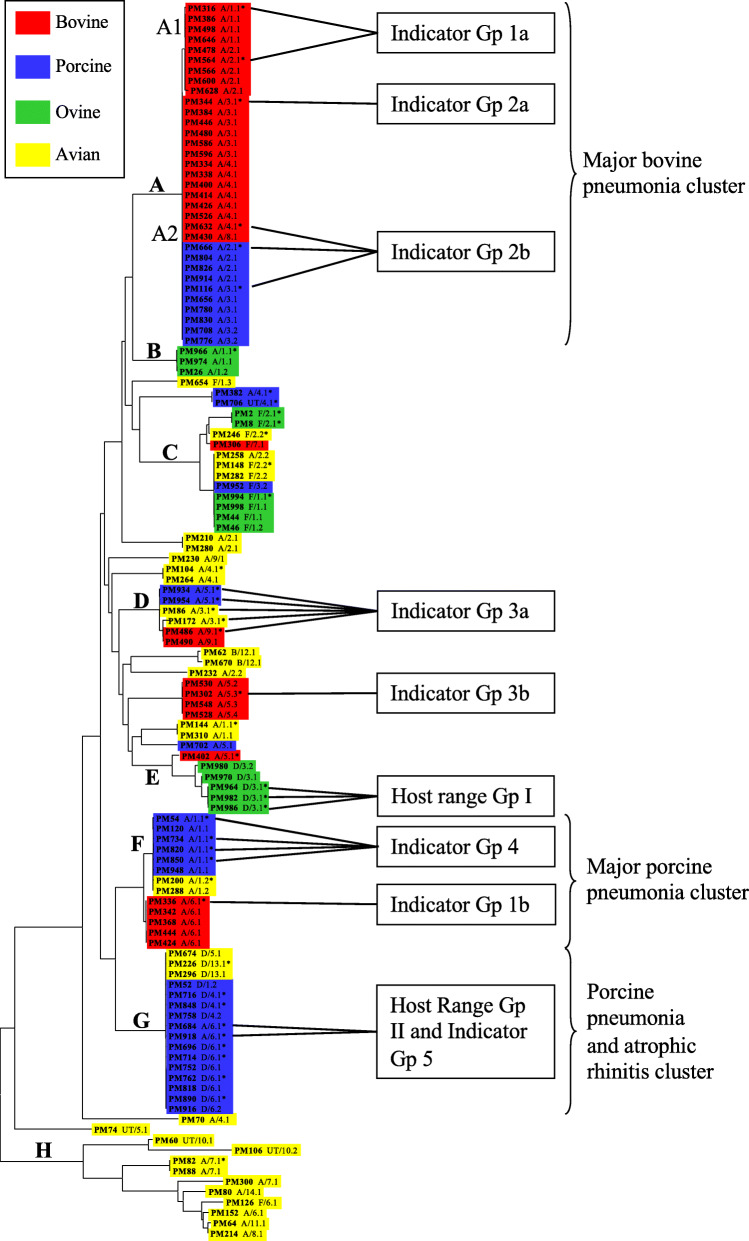
Phylogenetic relationships of 123 *P. multocida* isolates based on multilocus sequence typing (MLST). Neighbour-Joining tree representing the phylogenetic relationships of *P. multocida* strains based on the concatenated sequences (3990 bp) of seven housekeeping enzyme genes from 123 isolates of *P. multocida* of avian, bovine, ovine and porcine origin (http://pubmlst.org/pmultocida_multihost/). Isolate designations, capsular types and OMP types are provided for each isolate (e.g. PM316/A/1.1)

Phage DNA was successfully isolated from 18 of the 29 induced *P. multocida* cultures (Table 2). These isolates typically yielded large numbers of phage particles as observed by TEM. In most cases, a single band of varying intensity, demonstrating differing levels of phage induction between isolates, was identified (Fig. 3). However, two DNA bands of different molecular sizes were identified in the five isolates PM86, PM172, PM486, PM934 and PM954 suggesting the presence of at least two types of phage (Fig. 3). In preliminary RE experiments, the restriction profiles obtained with PsI, BamH1, Hind III, Ndel, EcoR1, XbaI were compared, and overnight incubation at 37 °C with HindIII was found to yield the most informative profiles (results not shown). DNA obtained from each of the 18 phage lysates was digested with HindIII and 10 different RE profiles, designated A to J, were identified (Fig. 4). The identification of 10 different RE types in lysates obtained from only 18 isolates demonstrated that the bacteriophages of *P. multocida* are relatively diverse. The association of the RE types with the various phage and isolates of origin is summarised in Table 2 and bacterial strain characteristics are summarised in Table 1. Type A was associated with *Myoviridae*-type phage from porcine isolate PM382 (capsular type A; ST13; MLST lineage C); type B was associated with *Myoviridae*-type phage from porcine isolates PM934 and PM954, bovine isolate PM486 and avian isolate PM172, (capsular type A; STs 15, 9 and 26; MLST lineage D); type C was associated with *Syphoviridae*-type phage from ovine isolates PM122, PM964, PM982, PM986 and PM988 (capsular type D; ST18; MLST lineage E); type D was associated with *Myoviridae*- and *Siphoviridae*-type phage from porcine isolate PM850 (capsular type A; ST10; MLST lineage F); type E was associated with *Siphoviridae*-type phage from bovine isolate PM336 (capsular type A; ST7; MLST lineage F); type F was associated with *Myoviridae*- and *Siphoviridae*-type phage from porcine isolate PM684 (capsular type A; ST11; MLST lineage G); type G was associated with *Siphoviridae*-type phage from porcine isolates PM918 and PM926 (capsular type A; ST11; MLST lineage G); type H was associated with *Myoviridae*-type phage from porcine isolate PM40 (capsular type A); type I was associated with *Siphoviridae*-type phage from porcine isolate PM848 (capsular type D; ST11; MLST lineage G) and type J was associated with *Myoviridae*- and *Siphoviridae*-type phage from avian isolate PM86 (capsular type A; ST15; MLST lineage D). These results demonstrated that certain RE types, such as B and C, were associated with phage induced in genetically identical or closely-related *P. multocida* isolates (i.e. MLST lineages D and E, respectively; Fig. 5). However, in other cases, phage of different RE types, such as F, G, and I, were present in closely related strains (MLST lineage G; Fig. 5) although these did differ in capsular type, OMP-type or disease syndrome (Table 1).

### Host range of induced phage and indicator strain sensitivities

**Fig. 6:**
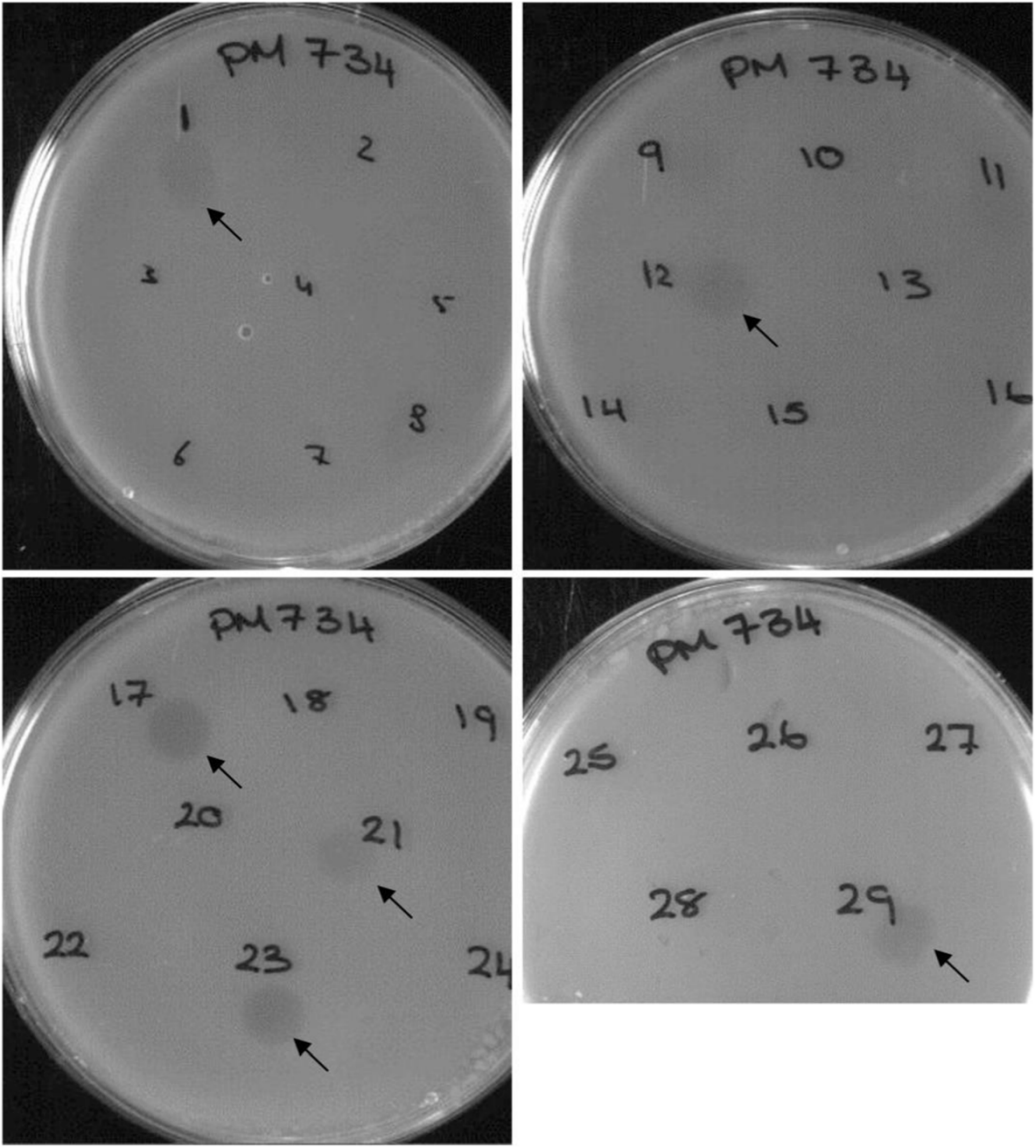
Plaque assay showing the activities of 29 induced lysates against isolate PM734 as an indicator strain. Faint lysis zones were produced by ФPM850 (1), ФPM964 (12), ФPM918 (21) and ФPM926 (29) (arrows), whereas ФPM684 (17) and ФPM40 (23) and produced clear lysis zones (arrows). The remaining lysates did not produce any signs of infection. Numbers (1 to 29) indicate phage (lysates) produced by 29 different *P. multocida* isolates. Indicator strain designations are shown at the top of each plate

The phage induced in the 29 *P. multocida* isolates were assessed for their abilities to infect indicator strains and to determine if there was any correlation between phage type and the ability to infect certain *P. multocida* strains. These experiments also provided information about the characteristics and relationships of strains that were sensitive to infecting phage. Spotting 10 μl of each lysate onto the assay plates resulted in the formation of zones of lysis with certain indicator strains after overnight incubation (Fig. 6). Of the 29 phage lysates, only 11 (38%) produced signs of infection; 18 (62%) lysates did not produce any visible signs of infection on any of the indicator strains used (Table 3). Differences were observed in the patterns of lysis produced by various lysates and the phage were accordingly assigned to phage “*lytic types*” or “*host range groups*” (HRGs) I, IIa, IIb, IIc, III and IV (Table 3). Similarly, differences were observed in the sensitivity of strains to lysis by the various phage, and sensitive strains were assigned to “*indicator strain groups*” (ISGs) 1a, 1b, 2a, 2b, 3a, 3b, 4 and 5.

**Table 3:** Host ranges of induced phages in 47 *P. multocida* indicator strains

Indicator strain^**a**^	Host species	MLST lineage	Capsular type	OMP-type	Indicator strain group (ISG)	Lytic type or host range group (HRG)^**b**^										
						I					II				III	IV
											a	b		c		
						**PM122**	**PM964**	**PM982**	**PM986**	**PM988**	**PM684**	**PM918**	**PM926**	**PM40**	**PM850**	**PM336**
PM316	Bovine	A	A	1.1	1a	±	±	±	±	±	±	±	±	±	±	-
PM564	Bovine	A	A	2.1	1a	±	±	±	±	±	±	±	±	±	+	-
PM344	Bovine	A	A	3.1	2a	+	+	+	+	+	-	-	-	-	-	-
PM632	Bovine	A	A	4.1	2b	+	+	+	+	+	+	+	+	+	-	-
PM666	Porcine	A	A	2.1	2b	+	+	+	+	+	+	+	+	+	-	-
PM116	Porcine	A	A	3.1	2b	+	+	+	+	+	+	+	+	+	-	
PM966	Ovine	B	A	1.1		-	-	-	-	-	-	-	-	-	-	-
PM382	Porcine	C	A	4.1		-	-	-	-		-	-	-	-	-	-
PM706	Porcine	C	UT	4.1		-	-	-	-	-	-	-	-	-	-	-
PM2	Ovine	C	F	2.1		-	-	-	-	-	-	-	-	-	-	-
PM8	Ovine	C	F	2.1		-	-	-	-	-	-	-	-	-	-	-
PM246	Avian	C	F	2.2		-	-	-	-	-	-	-	-	-	-	
PM994	Ovine	C	F	1.1		-	-	-	-	-	-	-	-	-	-	-
PM148	Avian	C	F	2.2		-	-	-	-	-	-	-	-	-	-	-
PM104	Avian	D	A	4.1		-	-	-	-	-	-	-	-	-	-	-
PM86	Avian	D	A	3.1	3a	±	±	±	±	±	-	-	-	-	-	-
PM934	Porcine	D	A	5.1	3a	±	±	±	±	±	-	-	-	-		-
PM954	Porcine	D	A	5.1	3a	±	±	±	±	±	-	-	-	-	-	-
PM486	Bovine	D	A	9.1	3a	±	±	±	±	±	+	-	-	-	-	-
PM172	Avian	D	A	3.1	3a	±	±	±	±	±	+	-	-	-	-	-
PM302	Bovine	E	A	5.3	3b	+	+	+	+	+	-	-	-	-	-	-
PM144	Avian	E	A	1.1		-	-	-	-	-	-	-	-	-	-	-
PM402	Bovine	E	A	5.1		-	-	-	-	-	-	-	-	-	-	-
PM122	Ovine	ND	D	3.1		-	-	-	-	-	-	-	-	-	-	-
PM964	Ovine	E	D	3.1		-	-	-	-	-	-	-	-	-	-	-
PM982	Ovine	E	D	3.1		-	-	-	-	-	-	-	-	-	-	-
PM986	Ovine	E	D	3.1		-	-	-	-	-	-	-	-	-	-	-
PM988	Ovine	ND	D	3.1		-	-	-	-	-	-	-	-	-	-	-
PM990	Ovine	ND	D	3.1		-	-	-	-	-	-	-	-	-	-	-
PM54	Porcine	F	A	1.1	4	-	-	-	-	-	+	+	+	+	-	-
PM734	Porcine	F	A	1.1	4	-	-	-	-	±	+	±	±	+	±	-
PM820	Porcine	F	A	1.1	4	-	-	-	-	-	+	+	+	+	-	-
PM850	Porcine	F	A	1.1	4	-	-	-	-	-	+	+	+	+	-	-
PM200	Avian	F	A	1.2		-	-	-	-	-	-	-	-	-	-	-
PM336	Bovine	F	A	6.1	1b	±	±	±	±	±	+	+	+	+	-	-
PM684	Porcine	G	A	6.1	5	+	+	+	+	+	-	-	-	-	-	-
PM918	Porcine	G	A	6.1	5	+	+	+	+	+	-	-	-	-	-	-
PM926	Porcine	ND	A	6.1	5	+	+	+	+	+	-	-	-	-	-	-
PM40	Porcine	ND	A	6.2	5	+	+	+	+	+	-	-	-	-	-	-
PM716	Porcine	G	D	4.1		-	-	-	-	-	-	-	-		-	-
PM848	Porcine	G	D	4.1		-	-	-	-	-	-	-	-	-	-	-
PM696	Porcine	G	D	6.1		-	-	-	-	-	-	-	-	-	-	-
PM714	Porcine	G	D	6.1		-	-	-	-	-	-	-	-	-	-	-
PM762	Porcine	G	D	6.1		-	-	-	-	-	-	-	-	-	-	-
PM890	Porcine	G	D	6.1		-	-	-	-	-	-	-	-	-	-	-
PM226	Avian	G	D	13.1		-	-	-	-	-	-	-	-	-	-	+
PM82	Avian	H	A	7.1		-	-	-	-	-	-	-	-	-	-	-

^a^ Indicator strains are arranged by order of MLST lineages (column 3, see also Fig. 5); ^b^ Host range group (HRG): (−) = no lysis, (±) = faint zone of lysis, (+) = clear zone of lysis

Lysates recovered from isolates PM122, PM964, PM982, PM986 and PM988 showed the broadest host range and represented HRG I. Notably, these phage were all *Siphoviridae* and of RE type C (Table 2) and were associated with closely-related ovine serotype D strains of OMP type 3.1 that represented a distinct cluster as part of lineage E on the phylogenetic tree (Table 3; Fig. 5). These phage caused lysis in 17 of 47 indicator strains and showed (with a single exception: PM964 lysate with indicator strain PM734) identical patterns of lysis (Table 3). The indicator strains that were sensitive to these phage lysates were represented by ISGs 1a (PM316, PM564), 1b (PM336), 2a (PM344), 2b (PM632, PM666, PM116), 3a (PM86, PM934, PM954 PM486, PM172), 3b (PM302), 4b (PM734) and 5 (PM684, PM918, PM926, PM40). Notably, there was a strong correlation between the strains representing each of these ISGs and the relatedness of the strains based on their phylogenies (Fig. 5), cell-surface characteristics (capsular serotype and OMP-types) and hosts of origin (Table 3). Thus, ISG 1a was associated with bovine serotype A isolates of lineage A1 whereas ISGs 2a and 2b comprised bovine and porcine serotype A isolates of lineage A2; ISG 3a was associated with a cluster of avian, bovine and porcine serotype A isolates that comprised lineage D; and ISG 5 consisted of porcine serotype A isolates of lineage G. Notably, these latter strains of ISG 5 are all *toxA*-containing strains and represent a cluster (lineage G) that includes a high proportion of strains associated with atrophic rhinitis (Table 1).

Lysates recovered from isolates PM684, PM918, PM926 and PM40 also showed a broad host range and represented HRG IIa (PM684), IIb (PM918, PM926) and IIc (PM40). Notably, phage from each of these groups were represented by RE types F, G and H, respectively, and were associated with closely-related porcine serotype A strains of OMP-type 6.1 (in the case of isolates PM684 and PM918) representing lineage G on the phylogenetic tree (Table 3; Fig. 5). These phage caused lysis in 10 of 47 indicator strains and showed similar, although not identical, patterns of lysis (Table 3). The indicator strains that were sensitive to these phage were represented by ISGs 1a (PM316, PM564), 1b (PM336), 2b (PM632, PM666, PM116), 4a (PM54, PM820, PM850) and 4b (PM734). Again, there was a strong correlation between the strains representing each of these ISGs and the relatedness of the strains based on their phylogenies (Fig. 5), cell-surface characteristics (capsular serotype and OMP-types) and hosts of origin (Table 3). The correlations for ISGs 1a and 2b have been described above; ISGs 4a and 4b were associated with porcine serotype A isolates of lineage F. Notably, these latter strains of ISG 4 belong to a cluster that is associated with porcine pneumonia (Fig. 5).

Lysates recovered from isolates PM850 and PM336 exhibited limited activity and represented HRGs III and IV, respectively. Interestingly, none of the phage lysates represented by RE type B (isolates PM934, PM954, PM486, PM172) were capable of lysing any of the indicator strains (Table 2). These phage were all of the *Myoviridae* type and were associated with the lineage D cluster of avian, bovine and porcine serotype A strains (Table 3, Fig. 5).

Overall, two observations among these results stood out above all others. First, it was highly significant that those nine isolates (PM122, PM982, PM986, PM988, PM964, PM684, PM918, PM926, PM40) representing HRGs I and II and with the broadest host ranges represent phylogenetically distinct ovine- and porcine-specific lineages and all contain the *toxA* gene (Table 1). Second, the possession of capsular type A was a feature common to all of the phage-sensitive indicator strains with a single exception; indeed there were only six serotype A strains that were insensitive to any of the phage lysates and none of the serotype D (with the single exception) or F strains exhibited sensitivity (Table 3). The single exception was an avian serotype D strain (PM226) that itself was sensitive to phage lysate from only a single isolate (PM336) (Table 3). Of particular relevance in this respect, closely related porcine strains of lineage G (Fig. 5) could be differentiated with respect to their capsular types and phage sensitivities; thus, serotype A strains were sensitive to phage of HRG 1 whereas serotype D strains were resistant.

## Discussion

*Pasteurella multocida* is an important veterinary pathogen causing different types of infection in various host species. Temperate bacteriophages are involved in generating bacterial diversity and driving the emergence of new pathogenic strains by disseminating genes encoding virulence factors, such as toxins, via the mechanism of horizontal gene transfer [18, 27–30]. The aim of the present study was to assess the presence and diversity of temperate bacteriophages in a well-characterized and diverse collection of *P. multocida* strains of known genetic relatedness recovered from avian, bovine, ovine and porcine host species.

Different concentrations of mitomycin C have previously been used to induce temperate bacteriophages in other bacterial species [41, 44, 45, 50–57] and we first set out to establish an optimum concentration for use with *P. multocida*. Eight isolates of avian, bovine, ovine and porcine origin (two each) and representing different disease syndromes, capsular serotypes, OMP-types and STs were selected for preliminary characterization. Initial experiments revealed that the effect of mitomycin C varied among the eight isolates with three different patterns being observed: complete lysis, partial lysis or reduced growth, and no lysis or no reduced growth (Fig. S1). Similar findings have been described previously in *E. coli* during the induction of *stx*_*2*_-converting bacteriophages with mitomycin C [44, 53]. These preliminary experiments allowed an optimum mitomycin C concentration of 0.2 μg/ml to be established for the induction of temperate bacteriophages in *P. multocida* isolates. Subsequent induction of the 47 *P. multocida* isolates with mitomycin C demonstrated that 15 (32%) isolates exhibited complete lysis, 22 (47%) were partially lysed or showed reduced growth, and 10 (21%) showed no lysis. However, since mitomycin C can influence bacterial growth in other ways, such as by the induction of bacteriocins, which may also lead to cell death and lysis [58], it was important to confirm phage induction visually by TEM or by the recovery of phage DNA.

Transmission electron microscopy has been extensively used as a tool in the study of bacteriophage morphology, characterization and classification [35, 50]. In the present study, temperate bacteriophages of different morphologies were identified by TEM in 29 *P. multocida* isolates. The phage particles identified belonged to the *Siphoviridae* (21 isolates) and *Myoviridae* (9 isolates) phage families although tail-less capsid particles (2 isolates) were also identified. A diverse set of temperate bacteriophages have previously been identified by TEM in *P. multocida* [35] as well as in the closely related species *M. haemolytica* [50]. Phage having diverse morphologies have also been identified in many other bacteria including *Burkholderia* [59]*, Clostridium* [55, 60], *Haemophilus* [42], *Lactobacillus* [61], *Listeria* [62], *Pseudomonas* [63], *Salmonella* [64] and *Yersinia* [51]. Overall, the results showed that phage particles of similar morphotypes were associated with closely related isolates (i.e. with the same, or closely related, genetic lineage). For example, isolates PM122, PM964, PM982, PM986 and PM988 of lineage E possessed phage of similar *Siphoviridae* morphotypes; isolates PM86, PM172, PM486, PM934 and PM954 of lineage D contained phage of similar *Myoviridae*-like morphotypes. In some isolates (e.g. PM86, PM684 and PM850), both *Siphoviridae-* and *Myoviridae-*type phage were induced from the same bacterial host, suggesting that a single *P. multocida* isolate may harbour multiple prophages. The presence of multiple phage in a single bacterial host has not previously been described in *P. multocida*, although this is known to occur in other bacteria including *M. haemolytica* [40, 56], *E. coli*, *Streptococcus pyogenes* and *Bacillus subtilis* [65]. The identification of temperate bacteriophages in 29 isolates suggests that these phage are likely to play important and widespread roles in the diversification and evolution of *P. multocida* since it is well established that bacteriophages play significant roles in bacterial evolution [19, 27, 32, 66].

Although phage particles were identified by TEM in 29 *P. multocida* lysates, phage DNA could only be isolated from 18 of the associated lysates. The concentration of DNA recovered varied from 50 to 200 ng/μl suggesting that some isolates have higher rates of phage production (phage replication and release) than others. Notably, phage yielded by isolates that underwent complete lysis produced the highest concentrations of DNA, whereas phage yielded by isolates that underwent partial lysis produced lower DNA concentrations. Such variation in the amount of DNA recovered from induced phage has also been described in *E. coli* [44, 53]. Classification of bacteriophages based on phage morphology has limitations because it does not provide information on genetic relatedness [67]. To overcome this problem, RE analysis of DNA has been used to assess the genetic diversity of induced phage in a wide-range of bacteria [36, 50, 53, 56, 60, 63, 68]. The identification of 10 different RE types in phage DNA recovered from only 18 isolates suggests that *P. multocida* bacteriophages are relatively diverse. As expected, multiple RE types were observed among phage having similar morphologies (i.e. *Myoviridae* or *Siphoviridae*) suggesting that phage of the same morphotypes are genetically divergent. Notably, RE types B, C and G were associated with two or more phage and phage of each RE type had identical morphologies (Table 2). Phage of RE type B had *Myoviridae*-type morphologies (and unusually small capsids), whereas phage of RE types C and G had *Siphoviridae*-type morphologies. Furthermore, phage representing each of these three RE types B, C and G were associated with closely-related isolates of lineages D, E and G, respectively (Fig. 5). Conversely, the association of more than one RE type with phage from isolates within the same lineage (e.g. RE types F [PM684], G [PM918, PM216] and I [PM848] of lineage G) suggests the presence of different phage types in closely related isolates; these isolates likely contain phage which have been acquired in recent phage-infection events. For example, isolate PM684 contains both *Myoviridae*- and *Siphoviridae*-type phage, whereas isolates PM918 and PM216 contain only a *Siphoviridae*-type phage, suggesting that the *Myoviridae*-type phage represents an additional acquisition. Clearly, further genomic analysis of the phage types associated with *P. multocida* is required to identify potential virulence genes that may be involved in the various disease syndromes caused by this pathogen.

Host range experiments were conducted using plaque assays to determine the presence of biologically active temperate bacteriophages and to assess the ability of induced phage to infect and lyse a range of indicator strains (Table 3). Overall, the plaque assays were less sensitive than TEM in determining the presence of phage in the lysates. Thus, of the 29 lysates that yielded phage particles (as determined by TEM), only 11 (38%) lysates produced signs of infection in indicator strains. The low number of phage able to infect the indicator strains may have been due to the low number of indicator strains possessing appropriate cell surface-associated phage receptors. Attachment of phage to host cells is dependent on the ability of phage proteins to recognise specific binding or attachment sites (receptors) on the bacterial cell surface [69, 70]. Such receptors include outer membrane proteins such as OmpA, porins and LamB, LPS, pili and flagella [69–76]. Very little is known about phage receptors of *P. multocida* and further studies are required to identify these. Bacteriophages use different mechanisms to infect encapsulated bacteria and reach the bacterial cell surface because the capsule may block bacteriophage access to receptors localised in the cell wall [69, 70]. Some phage produce capsular depolymerase enzymes that cause degradation of the capsular layer and allow the phage to reach the bacterial cell wall [69]. Therefore, it is probably significant that all but one of the phage-susceptible isolates was of capsular type A (Table 3). This capsular type might be acting directly as the phage receptor or it might be susceptible to a phage-generated capsular depolymerase. The major component of the type A capsule is hyaluronic acid [77] and the type A capsule is known to be sensitive to hyaluronidase activity [78]. Thus, it would be interesting to speculate that many of these phage may possess hyaluronidase activity. Conversely, these observations might also reflect differences in phage tail fibre structure, or the complete absence of tail fibres, since the tail fibres are involved in the attachment of phage to receptors present on the bacterial cell surfaces [69, 70]. Alternatively, some of these phage may have infected certain isolates and become incorporated into the bacterial genomes without causing lysis of these strains. In support of the latter hypothesis, temperate bacteriophages induced in Shiga-toxin producing *E. coli* were not detectable directly by plaque assay although these phage were detected by hybridisation [53]. Induced phage may also be unable to produce zones of lysis because certain prophages confer immunity via repressor proteins [56, 73].

Of the 11 phage lysates that caused infection of indicator strains, nine had relatively broad host ranges, infecting from 10 to 18 isolates (Table 3). These phage comprised two groups (HRGs I and II) which could be distinguished by their origins. Phage lysates of HRG I comprised *Siphoviridae*-type phage of RE type C which originated from ovine serotype D strains of lineage E. Phage lysates of HRG II were more diverse, representing three sub-groups, IIa, IIb and IIc; lysates of HRGs IIa, IIb and IIc comprised *Myoviridae*- and/or *Siphoviridae*-type phage of RE types F, G and H, respectively (Table 2). Phage of HRG II had similar origins in that they were recovered from porcine serotype A strains of lineage G (Fig. 5). However, the most notable feature of these nine phage was that they all originated from ovine or porcine strains that possessed the *toxA* gene (Table 1). Pullinger et al. [36] demonstrated that *toxA* is associated with *Siphoviridae*-type phages in *P. multocida* and it is interesting to speculate that it is, perhaps, *toxA*-containing *Siphoviridae*-type phages that are responsible for the lysis of the indicator strains described herein. Indeed, subsequent genomic analysis of the phage DNA within these strains has shown that isolates PM122, PM964, PM982, PM986 and PM988 contain identical lambda-like *Siphoviridae* phage which are genetically distinct from identical lambda-like phage in isolates PM684, PM918, PM926 and PM940 (results not shown). Significantly, all nine phage sequences also contain the *toxA* gene. The non-identity of these two phage groups also correlates with the differing host ranges of the phage lysates (Table 3). Thus, phage of HRG I lysed bacteria of ISGs 1a, 1b, 2a, 2b, 3a, 3b and 5 whereas phage of HRG II lysed bacteria of ISGs 1a, 1b, 2b, 4a and 4b. Interestingly, none of these phage infected the same strains from which they originated, although phage of HRG I did infect the strains from which phage of HRG II were derived (Table 3).

Finally, five of the nine *Myoviridae*-type phage identified in the current study were unusual in that they all had a very distinct morphological appearance and were quite different from the other *Myoviridae*-type phage observed. These phage particles had unusually small hexagonal capsids and long tails compared to the other *Myoviridae*-type phage (Fig. 2f; Table 2). This phage type was induced exclusively in isolates PM86, PM172, PM486, PM934 and PM954 which belong to the same MLST cluster (i.e. lineage D, Fig. 5); isolates of this cluster were recovered from avian, bovine and porcine hosts. This type of *Myoviridae*-like phage, characterised by a small capsule and long tail, has not previously been described in *P. multocida* although phage of similar morphology have been identified in *Clostridium difficile* 027 strains [55] and in *Bacillus* species [79]. Phage particles with small capsids have also been identified as components of *Staphylococcus aureus* pathogenicity islands (SaPIs) [80]. SaPIs are phage-inducible chromosomal islands (PICIs) which maintain an intimate relationship with temperate helper bacteriophages. Following induction of the SOS response, the SaPI genome excises, replicates using its own replicon and encapsidates into small phage heads (to fit their smaller genome) produced by the helper phage [80, 81]. The identification of *Myoviridae*-type phage particles with unusually small heads, together with the presence of two DNA bands representing two phage genomes of differing size (Fig. 3), strongly suggests the presence and induction of PICIs in *P. multocida* isolates PM86, PM172, PM486, PM934 and PM954. Indeed, this observation represents the first description of PICIs in *P. multocida* which has been confirmed elsewhere [82]. The exclusive association of PICIs and helper phage with closely-related isolates of lineage D suggests that they have been acquired, most-likely by an ancestral strain, from an external source prior to the dissemination of these isolates to different host species.

## Conclusions

The majority (79%) of *P. multocida* isolates were sensitive to mitomycin C indicating that these isolates contain temperate bacteriophages. TEM subsequently identified a diverse set of phage in 29 induced cultures. The phage particles were morphologically diverse and represented the *Siphoviridae* and *Myoviridae* families. Both *Siphoviridae*- and *Myoviridae*-type phage were induced in certain isolates indicating that a single host may harbour multiple prophages. Moreover, a distinct *Myoviridae* phage type with an unusually small capsid was identified in certain isolates. The identification of 10 different RE profiles in the DNA of only 18 phage provided evidence that temperate bacteriophages in *P. multocida* are relatively diverse. Plaque assays were less sensitive than TEM for detection of temperate bacteriophages. Only 11 (38%) phage lysates produced signs of infection against indicator strains and bacterial phage sensitivity was almost exclusively associated with strains of capsular type A. However, the majority (9/11) of phage lysates which caused infection originated from two groups of phylogenetically unrelated ovine and porcine strains that uniquely possessed the *toxA* gene.

## Materials and methods

### Bacterial isolates and growth conditions

Forty-seven *P. multocida* isolates, obtained from regional laboratories of the UK Veterinary Laboratories Agency (VLA), were selected for characterization of their temperate bacteriophages. The isolates were recovered from cattle, sheep, pigs and poultry and were associated with different disease syndromes. They were selected to represent specific capsular serotypes types, OMPs-types and multilocus STs (Fig. 5) and have been well-characterised in previous studies [9, 46–49]. The properties of the isolates are summarised in Table 1. The isolates were stored in 50% (v/v) glycerol in brain heart infusion broth (BHIB; Oxoid) at − 80 °C and cultured on blood agar (brain heart infusion agar [BHIA; Oxoid] containing 5% [v/v] defibrinated sheep’s blood overnight at 37 °C. Liquid cultures were prepared by resuspending 3 to 4 well-isolated colonies (from an overnight plate culture) in 3 ml BHIB in a bijoux. Unless otherwise stated, 0.3 ml of this suspension were used to inoculate 30-ml pre-warmed BHIB in a 100 ml Erlenmeyer flask which was incubated at 37 °C with shaking at 120 rpm.

### Induction and preparation of phage suspensions

Bacteriophages were induced using a modification of a previously published procedure [50]. A final optimal concentration of 0.2 μg/ml mitomycin C (Sigma-Aldrich, UK) was determined in preliminary experiments using a panel of eight *P. multocida* strains. Briefly, two 30 ml cultures of each isolate were inoculated and incubated as described above. Mitomycin C was added to one of each pair of flasks when the optical density (OD_600_) reached 0.4 and the cultures were incubated for a further 8 h. The rate of bacterial cell lysis (induction) was monitored by measuring the OD_600_ for both the mitomycin C-treated and control cultures at hourly intervals. For those isolates which showed no signs of lysis after 8 h, incubation was continued for 24 h. In the absence of any signs of induction (i.e. no visible clearing in a specific isolate even after 24 h), the experiment was repeated using higher concentrations of mitomycin C (i.e. 0.5, 1.0 and 2.0 μg/ml). Bacteriophage induction was performed in triplicate for each isolate. The induction profile of each isolate was assigned (complete, partial or none) by comparing the OD_600_ values of mitomycin C-treated and control cultures over the period of incubation. Phage suspensions were prepared by centrifuging the induced cultures at 4000 x g for 20 min at 4 °C to remove unlysed bacterial cells. The supernatants were carefully removed and filtered using 0.2 μm pore-size syringe filters (Minisart) to remove bacterial debris. Filtered phage suspensions were either used immediately for host range determination or stored at 4 °C for further characterization and analysis.

### Transmission electron microscopy

Ten millilitres of each filtered phage suspension were centrifuged at 40,000 x g for 90 min at 4 °C. The supernatants were carefully removed and the pellets re-suspended gently in 0.5 ml of 0.1 M ammonium acetate (pH 7.3). The suspensions were stored overnight at 4 °C for negative staining and vizualisation by TEM. Three hundred-mesh carbon-coated nickel grids were dropped onto 50–100 μl of phage suspension. The grids were allowed to adsorb phage suspension for 1–2 min and were washed three times with dH_2_O for 10 s. Excess fluid was removed using Whatman filter paper and the grids were negatively stained with 2% ammonium molybdate for 30 s. Excess staining solution was removed with Whatman filter paper and the grids allowed to air-dry at room temperature for 15 to 20 min. The samples were examined by TEM using a FEI Tecnai TF20 electron microscope at 200 kV and Gatan Microscopy Suite Software.

### Phage purification and DNA extraction

To obtain sufficient numbers of phage particles for DNA extraction, bacteria were grown in 50 ml volumes of BHIB in 250 ml Erlenmeyer flasks prior to induction of bacteriophages. After induction, the bacteria were removed by centrifugation and filtration as described above and the filtered lysates stored at 4 °C. Phage DNA was obtained by the standard method described for bacteriophage lambda DNA [83]. Briefly, 40 ml of filtered lysate were transferred into sterile 50 ml centrifuge tubes and allowed to reach room temperature. DNase (Sigma-Aldrich, UK, Cat.No.D4263) and RNase (Sigma-Aldrich, UK, Cat.No. R4642) were added to final concentrations of 10 μg/ml and incubated for 1 h at 37 °C. Solid NaCl (Fisher Scientific, UK) was added to a final concentration of 1 M. The mixture was dissolved and left to stand for 1 h on ice. Bacterial debris was removed by centrifugation at 11,000 x g for 10 min at 4 °C and the supernatants collected into clean centrifuge tubes. The phage particles were concentrated by adding polyethylene glycol (PEG 8000; Sigma-Aldrich, UK) to a final concentration of 10% (w/v). The PEG 8000 was slowly dissolved by stirring and the treated mixture allowed to stand on ice for 1 h. Precipitated phage particles were recovered by centrifugation at 11,000 x g for 15 min at 4 °C. The supernatants were carefully removed and the tubes left to dry for 10 min at room temperature. Finally, the pelleted phage particles were re-suspended in 200 μl phage buffer (0.1 M MgSO_4_, 1 M CaCl_2_, 2.95 g of NaCl, 2.5 M Tris, pH 8) and stored at 4 °C for DNA extraction. To each aliquot of re-suspended phage, 200 μl of lysis mix were added (19 μl of 20% SDS [Sigma-Aldrich, UK], 9 μl of proteinase K [20 mg/ml; Promega] and 172 μl of dH_2_O) and the mixture incubated at 56 °C for 1 h. Phenol-chloroform-isoamyl alcohol (25:24:1; Sigma-Aldrich, UK) extraction and ethanol precipitation were used to extract the phage DNA as previously described [83]. The DNA was recovered by centrifugation at 13,000 x g for 1 min at 4 °C and its concentration measured using a NanoDrop 2000C spectrophotometer (Thermo Scientific). Purified phage DNA was separated by agarose gel electrophoresis and visualised with Syber® Safe DNA gel stain (Invitrogen). The DNA was stored at − 20 °C.

### Restriction endonuclease analysis

The genetic relatedness of the phage induced in *P. multocida* was assessed by RE analysis using the restriction enzymes PsI, BamH1, Hind III, Ndel, EcoR1 and XbaI (New England Biolabs Ltd., UK) according to the manufacturer’s instructions. Briefly, the final 20 μl reaction mixtures contained DNA (1 μg/μl), restriction enzyme (1 μl) and cut smart buffer (2 μl), and the reactions were incubated at 37 °C for 4 h. In some experiments digestion was performed overnight at 37 °C. Digested phage DNA was separated by agarose gel electrophoresis and visualised with Syber® Safe DNA gel stain (Invitrogen). The size of the DNA bands was estimated using a 1 kb plus DNA ladder (Invitrogen).

### Bacteriophage host range

The ability of induced phage to infect *P. multocida* indicator strains was assessed using the double agar overlay plaque assay. The host range of phage induced in 29 isolates was examined against all 47 *P. multocida* isolates. The indicator strains were grown overnight in BHIB at 37 °C with shaking at 120 rpm. Overlay plates were prepared using a base layer of 1% BHIA and a top layer of soft agar comprising BHIB, 0.7% (w/v) Microagar (Duchenne, London, UK) and 2 mM CaCl_2_ (PROLABO). The semi-solid medium (BHIB + 0.7% microagar) and CaCl_2_ (1 M) were autoclaved separately at 120 °C for 15 min to prevent precipitation. The semi-solid medium was cooled to 56 °C and the CaCl_2_ (1 M) added to a final concentration of 2 mM. The molten soft agar was held in a water bath at 48 °C to avoid solidification. Bacterial lawns were prepared by adding 50 μl of broth cultures to 3 ml of warm molten soft agar in a bijoux. Bacteria and agar were mixed and poured immediately onto the surface of the dried base layers with gentle rocking. The top layers were allowed to solidify and dried at room temperature for 15 to 20 min. Ten microlitres of each filtered phage suspension were spotted onto dried plates seeded with each of the 47 indicator strains. The plates were allowed to dry for 20–30 min and incubated at 37 °C for 24 h before assessment of bacterial lysis. The interaction of each phage/indicator strain combination was assessed in triplicate.

## Supplementary Information

**Additional file 1.**

## Data Availability

Not applicable.
