# Supplementary material for: Diversity and characterization of temperate bacteriophages induced in Pasteurella multocida from different host species
Source: BMC Microbiol. 2021 Mar 30;21:97. doi: 10.1186/s12866-021-02155-9 (PMC8008546; doi:10.1186/s12866-021-02155-9)
Supplement: Supplementary file 1 — Additional file 1. [file 12866_2021_2155_MOESM1_ESM.pdf]

Diversity and characterization of temperate bacteriophages induced in *Pasteurella multocida* from different host species

Rezheen F. Abdulrahman[1,2] and Robert L. Davies[1*]

[1]*Institute of Infection, Immunity and Inflammation, College of Medical, Veterinary and Life Sciences, Sir Graeme Davies Building, University of Glasgow, Glasgow G12 8TA, UK;* [2]*Pathology and Microbiology Department, University of Duhok, Kurdistan Region, Iraq*

**Corresponding author email: Robert.Davies@glasgow.ac.uk*

**Corresponding author address:*

Dr. Robert L. Davies

Institute of Infection, Immunity and Inflammation

College of Medical, Veterinary and Life Sciences

Sir Graeme Davies Building

University of Glasgow

120 University Place

Glasgow G12 8TA

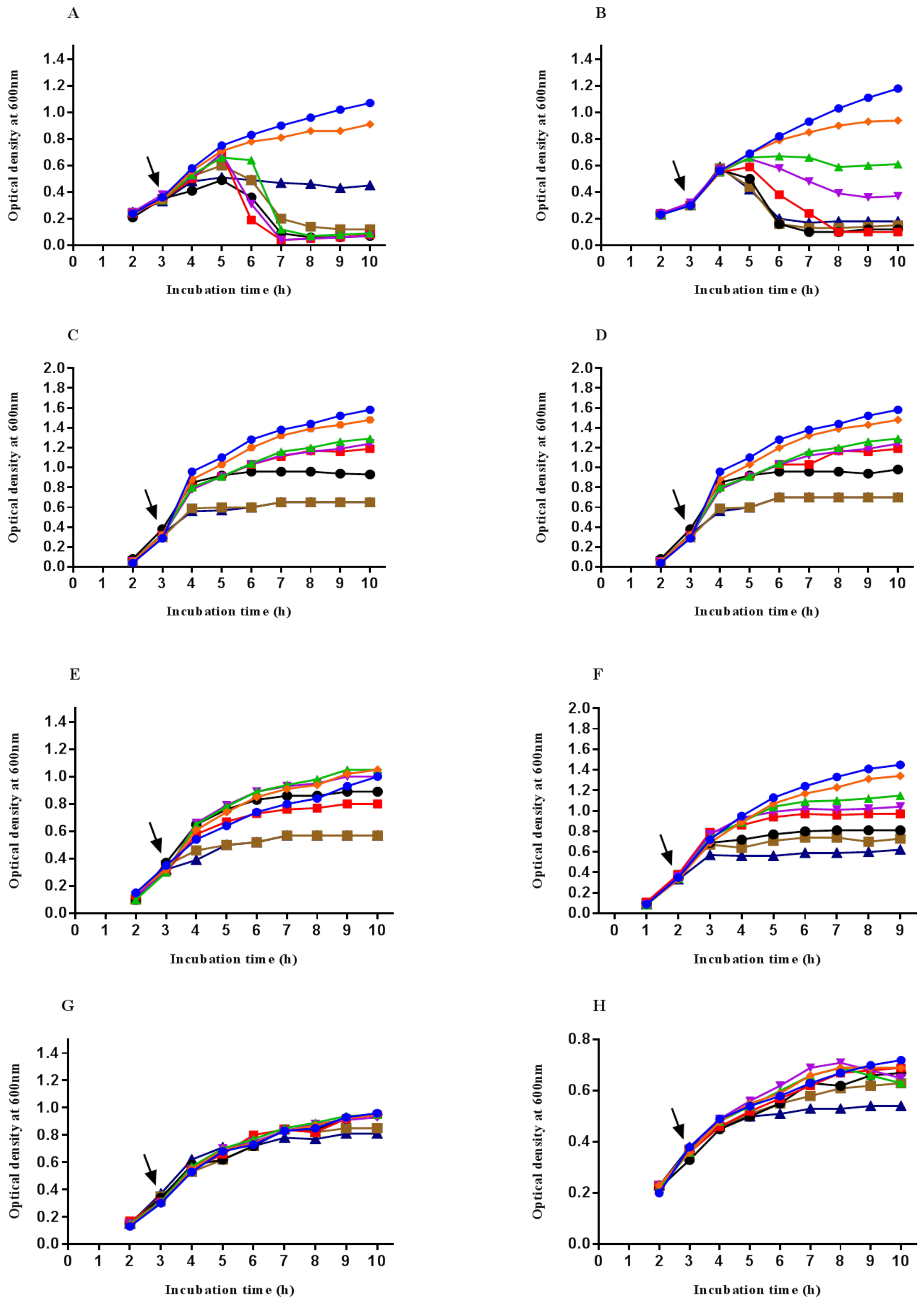

Fig. S1 Induction profiles comparing different mitomycin C concentrations.

Isolates PM684 (A), PM982 (B), PM246 (C), PM632 (D), PM734 (E), PM966 (F), PM144 (G) and PM564 (H) were used to compare different mitomycin C concentrations. The following concentrations of mitomycin C were used for each isolate: 0 (●), 0.01 (◆), 0.05 (▲), 0.1 (▼), 0.2 (■), 0.5 (●), 1.0 (■) and 2.0 (▲) µg/ml. The figure shows three different patterns of lysis: complete (A & B) partial (C, D, E, & F) and no lysis (G & H). The arrows indicate the point at which the mitomycin C was added. Graphs were created using GraphPad Prism 7.

Table S1 Induction profile in 47 *P. multocida* isolates.

Isolate[a]	Host species	Capsular type	OMP-type[b]	ST[c]	MLST lineage	*toxA* gene	Mitomycin C	Lysis type[d]	Phage type[f]
PM200	Avian	A	1.2	10	F	ND	2 µg/ml	Complete	S
PM336	Bovine	A	6.1	7	F	ND	0.2 µg/ml	Complete	S
PM122	Ovine	D	3.1	ND	ND[e]	+	0.2 µg/ml	Complete	S
PM964	Ovine	D	3.1	18	E	+	0.2 µg/ml	Complete	S
PM982	Ovine	D	3.1	18	E	+	0.2 µg/ml	Complete	S
PM986	Ovine	D	3.1	18	E	+	0.2 µg/ml	Complete	S
PM988	Ovine	D	3.1	ND	ND	+	0.2 µg/ml	Complete	S
PM382	Porcine	A	4.1	13	C	-	0.2 µg/ml	Complete	M
PM850	Porcine	A	1.1	10	F	-	0.2 µg/ml	Complete	S&M
PM684	Porcine	A	6.1	11	G	+	0.2 µg/ml	Complete	S&M
PM918	Porcine	A	6.1	11	G	+	0.2 µg/ml	Complete	S
PM926	Porcine	A	6.1	ND	ND	+	0.2 µg/ml	Complete	S
PM40	Porcine	A	6.2	ND	ND	+	0.2 µg/ml	Complete	M
PM716	Porcine	D	4.1	11	G	+	0.2 µg/ml	Complete	-
PM848	Porcine	D	4.1	11	G	+	0.2 µg/ml	Complete	S
PM246	Avian	F	2.2	25	C	ND	2 µg/ml	Partial	-
PM86	Avian	A	3.1	15	D	ND	0.2 µg/ml	Partial	S&M
PM172	Avian	A	3.1	26	D	ND	0.2 µg/ml	Partial	M
PM226	Avian	D	13.1	11	G	-	0.5 µg/ml	Partial	S
PM344	Bovine	A	3.1	3	A	ND	1 µg/ml	Partial	-
PM632	Bovine	A	4.1	4	A	ND	2 µg/ml	Partial	-
PM486	Bovine	A	9.1	9	D	ND	0.2 µg/ml	Partial	M
PM402	Bovine	A	5.1	5	D	ND	1 µg/ml	Partial	-
PM666	Porcine	A	2.1	3	A	-	0.2 µg/ml	Partial	S
PM116	Porcine	A	3.1	3	A	-	2 µg/ml	Partial	S
PM966	Ovine	A	1.1	16	B	-	2 µg/ml	Partial	S
PM990	Ovine	D	3.1	ND	ND	+	0.2 µg/ml	Partial	-
PM706	Porcine	UT	4.1	13	C	-	0.2 µg/ml	Partial	-
PM934	Porcine	A	5.1	15	D	-	0.2 µg/ml	Partial	M
PM954	Porcine	A	5.1	15	D	-	0.2 µg/ml	Partial	M
PM54	Porcine	A	1.1	10	F	-	0.2 µg/ml	Partial	S
PM734	Porcine	A	1.1	10	F	-	2 µg/ml	Partial	-

Table S1 (*continued*)

Isolate[a]	Host species	Capsular type	OMP-type[b]	ST[c]	MLST lineage	*toxA* gene	Mitomycin C	Lysis type[d]	Phage type[f]
PM820	Porcine	A	1.1	10	F	-	0.2 µg/ml	Partial	S
PM696	Porcine	D	6.1	11	G	+	0.2 µg/ml	Partial	S
PM714	Porcine	D	6.1	11	G	-	0.2 µg/ml	Partial	S
PM762	Porcine	D	6.1	11	G	+	2 µg/ml	Partial	-
PM890	Porcine	D	6.1	11	G	+	2 µg/ml	Partial	-
PM148	Avian	F	2.2	12	C	ND	0.2 µg/ml	No lysis	-
PM104	Avian	A	4.1	28	D	ND	0.2 µg/ml	No lysis	-
PM144	Avian	A	1.1	21	E	ND	0.2 µg/mL	No lysis	-
PM82	Avian	A	7.1	32	H	ND	0.2 µg/ml	No lysis	-
PM316	Bovine	A	1.1	1	A	ND	0.2 µg/ml	No lysis	-
PM564	Bovine	A	2.1	1	A	ND	0.2 µg/ml	No lysis	-
PM302	Bovine	A	5.3	6	E	ND	0.2 µg/ml	No lysis	-
PM2	Ovine	F	2.1	17	C	-	0.2 µg/ml	No lysis	-
PM8	Ovine	F	2.1	17	C	-	0.2 µg/ml	No lysis	-
PM994	Ovine	F	1.1	12	C	-	0.2 µg/ml	No lysis	-

[a] Isolates are arranged by order of MLST lineage (column 6; Fig. 5). [b] OMP-types for bovine, ovine, porcine and avian isolates have been defined previously [9, 46–49] and are not equivalent, i.e. bovine OMP-type 1.1 is not as same as porcine OMP-type 1.1, etc. [c] ST: sequence type (Davies *et al.*, unpublished; http://pubmlst.org/pmultocida_multihost). [d] Lysis types are defined in the text (see Fig. 1). [e] ND: not determined. [f] Phage type based on TEM (S: *Siphoviridae*; M: *Myoviridae*) and tail-less capsids were identified in PM762 and PM890.
